# Fetoscopic myelomeningocele repair: standard technique and approaches for closure of large defects

**DOI:** 10.3171/2026.1.FOCVID25226

**Published:** 2026-04-01

**Authors:** Arjun R. Adapa, Anthony J. Tang, Nicholas Schmoke, Lynn Simpson, Russell Miller, Laurence Ring, Vincent P. Duron, Neil A. Feldstein

**Affiliations:** 1 Department of Neurological Surgery, Columbia University Irving Medical Center, New York;; 2 Division of Pediatric Surgery, Columbia University Irving Medical Center, New York;; 3 Department of Obstetrics and Gynecology, Columbia University Irving Medical Center, New York; and; 4 Division of Obstetric Anesthesia, Department of Anesthesia, Columbia University Irving Medical Center, New York, New York

**Keywords:** fetoscopic myelomeningocele repair, spina bifida, myeloschisis, large defect closure, prenatal surgery, fetal neurosurgery

## Abstract

Fetoscopic repair of myelomeningocele (MMC) offers a minimally invasive alternative to open prenatal surgery. This approach protects exposed neural tissue while reducing the need for postnatal CSF diversion and lowering maternal morbidity compared to open techniques. In this video, the authors demonstrate a standardized fetoscopic technique for MMC repair using an externalized uterine approach. Key steps include placode release, excision of transitional epithelium, and multilayer closure with a dural substitute. The authors highlight technical pearls for managing myeloschisis and large MMCs, including relaxing incisions and myofascial mobilization, to achieve a tension-free, watertight repair.

The video can be found here: https://stream.cadmore.media/r10.3171/2026.1.FOCVID25226

## Transcript

This video highlights fetoscopic myelomeningocele repair.

### 0:24 Myelomeningocele Background.

Fetal myelomeningocele (MMC) is a neural tube defect in which the spinal cord remains exposed, leading to progressive neurological injury throughout gestation.^1–4^ Myelomeningocele has a prevalence of 3 cases for every 10,000 births in the US and is associated with significant postnatal disability.^5^ It is the most common congenital defect of the central nervous system.^3^ Most fetuses with myelomeningocele have Chiari II malformation and are at high risk for hydrocephalus, often requiring ventriculoperitoneal shunting with significant morbidity.^4,6^

### 0:50 Rationale for Prenatal Repair and Fetoscopic Repair.

As shown in the MOMS trial, prenatal repair reduces shunt rates and improves motor outcomes compared with postnatal surgery.^5^ Fetoscopic repair provides these benefits while reducing maternal morbidity associated with open hysterotomy.^7^

### 1:02 Multidisciplinary Team.

At our institution, the fetoscopic repair is done with a multidisciplinary team, which includes pediatric surgery, maternal-fetal medicine, pediatric neurosurgery, OB anesthesia, nursing, and an intraoperative sonographer.

### 1:15 Methods.

In addition to demonstrating surgical technique, this video includes a preliminary summary of institutional outcomes. Outcomes were derived from a prospective, single-center review of all fetoscopic myelomeningocele repairs from May 2019 through July 2025. Candidates were selected using the MOMS trial eligibility criteria.^5^

Preoperative evaluation includes detailed fetal imaging to document lesion level and morphology, confirm Chiari II malformation, and measure ventricular size.

### 1:45 Representative Prenatal Imaging.

An example of a prenatal MR fetus is shown, which shows classic features associated with myelomeningocele, including concavity of the frontal bones, or the "lemon sign" on the left, dilated lateral ventricles, and Chiari II malformation.

### 2:00 Preoperative Preparation.

Patients are admitted the night before surgery to complete preoperative labs, evaluation of maternal stability, and anesthesia assessment. On the day of the procedure, an epidural is placed and general anesthesia is induced. A Pfannenstiel incision with midline fascial incision is performed, and the uterus is externalized. Amnioinfusion is performed, and stay sutures are placed around the intended port site in a box configuration. The first port is placed under ultrasound guidance using the Seldinger technique. After amnioreduction, the uterus is insufflated with carbon dioxide. Two additional ports are placed under direct visualization.

### 2:38 Fetal Cocktail Administered.

A standard intramuscular fetal cocktail consisting of vecuronium and fentanyl is administered.

### 2:44 Stay Suture Placement.

If indicated, a stay suture is then placed either cephalad or caudal to the lesion in order to help stabilize the fetus. Potential but low risks include localized bleeding or skin injury at the entry point and a theoretical risk of membrane disruption, which we mitigate through careful placement under endoscopic visualization.

### 3:08 Initial Dissection.

Dissection begins circumferentially along the margins of the placode.

### 3:14 Lateral Dissection of Placode.

Dissection proceeds along the lateral edges of the placode to free neural tissue.

### 3:20 Placode Released.

The neural placode is carefully released from surrounding tissues. Placode imbrication is not performed in our fetoscopic technique. While some postnatal repairs may include imbrication under open exposure, we prioritize atraumatic handling and efficient multilayer closure in utero due to tissue fragility and limited working space.

### 3:53 Mobilization of Skin Flaps.

Skin flaps are mobilized to prepare for tension-free closure.

### 4:00 Excision of Transitional Epithelium.

Transitional epithelium is excised. When hemostasis is required, monopolar cautery scissors are used sparingly at a low-power setting of 10 W for superficial bleeding points away from the placode. Tamponade and irrigation are preferred whenever possible, and direct contact with neural tissue is avoided.

### 4:20 DuraGen Patch Placement.

A DuraGen collagen matrix is placed as an overlay dural substitute and is not sutured to native dura in our technique. Watertightness is achieved through subsequent multilayer closure.

### 4:31 Central Tension-Relieving Stitch.

A central stitch is then placed to approximate the skin edges and relieve tension prior to final closure.

### 4:40 Running V-Loc Closure.

The skin is closed using a running 4-0 V-Loc suture, creating a watertight repair.

### 5:02 Postnatal Images.

Here we show postoperative images from days of life 1 and 3, demonstrating durable, watertight closure with no cerebrospinal fluid leak.

### 5:12 Myeloschisis Repair.

In the second clip, a myeloschisis defect is demonstrated, highlighting techniques for large defects with limited skin mobility.

### 5:19 Lateral Placode Dissection.

Dissection is performed along the lateral margins to fully release the placode.

### 5:46 Placode Released.

Circumferential release is achieved.

### 5:49 Limited Skin Flap Mobility.

Skin flaps exhibit limited medial mobility, precluding primary closure.

### 5:55 Skin Edges Lateral to Placode.

The skin edges lie significantly lateral relative to the placode.

### 6:01 Indication for Relaxing Incision.

A relaxing incision is required to achieve a tension-free closure.

### 6:05 Flank Relaxing Incision.

A relaxing incision is made along the flank to improve tissue mobility.

### 6:14 Dissection to Muscle and Fascia.

The dissection proceeds down to the muscle and fascial layers.

### 6:23 Completed Relaxing Incision.

The relaxing incision is complete.

### 6:25 Patch Placement.

A dural substitute patch is placed over the exposed placode.

### 6:31 Myofascial Approximation.

Interrupted sutures are used to approximate the myofascial layer.

### 6:45 Skin Closure with V-Loc Maxon.

Skin is closed using a 4-0 V-Loc Maxon suture in a running fashion.

### 6:52 Completed Repair and Postnatal Images.

A tension-free, multilayer closure is completed. Postop images from days of life 1 and 9 demonstrate durable closure.

### 7:02 Large Myelomeningocele Repair.

The final clip demonstrates repair of a large myelomeningocele defect. The neural placode is circumferentially released. Dissection is initiated at the superior aspect of the placode, which is handled gently using atraumatic instruments and no traction. A moist operative field and continuous endoscopic visualization are maintained to protect the ascending spinal cord and vascular arcade.

### 7:24 Excision of Transitional Epithelium.

Transitional epithelium is excised.

### 7:30 Elevation of Skin Flaps.

Skin flaps are elevated and mobilized to facilitate closure.

### 7:41 Mobilization of Myofascial Layer.

The myofascial layer is mobilized to create an additional reconstructive layer.

### 7:55 DuraGen Patch Placement.

A DuraGen collagen matrix is placed over the placode.

### 8:00 Myofascial Closure With Interrupted Vicryl.

Interrupted Vicryl sutures are used to close the myofascial layer.

### 8:18 Skin Closure.

Skin closure is completed using horizontal mattress and interrupted sutures.

### 8:54 Completed Multilayer Repair and Postnatal Image.

A stable, watertight multilayer repair is achieved. A postoperative image from day of life 4 demonstrates durable closure.

### 9:02 Postnatal Care.

Postnatal care is coordinated through a multidisciplinary spina bifida clinic involving pediatric surgery, neurosurgery, neurology, physiatry, and urology.

### 9:10 Early Institutional Outcomes.

Early outcomes from our first 17 patients demonstrate excellent fetal neurological results. At the time of video preparation, the average patient age is approximately 3 years, with all but 1 patient followed for more than 1 year. Our 12-month VP shunt rate is 12%, compared with 40% after open repair in the MOMS trial and 42% reported in a meta-analysis of fetoscopic studies.^5,8^ Furthermore, 82% of patients in our cohort exhibited reversal of hindbrain herniation, compared to the 36% rate in the MOMS trial. Maternal outcomes in our cohort were also favorable, with no cases of uterine dehiscence or placental abruption.

### 9:46 Example Postnatal MRI.

An example of a postnatal MRI is shown at 1 year of life, which demonstrated reversal of hindbrain herniation.

### 9:53 Conclusions.

In conclusion, fetoscopic myelomeningocele repair using an externalized uterine technique provides a minimally invasive alternative to open prenatal surgery and is a safe option for carefully selected patients. Tailored strategies, including relaxing incisions and myofascial mobilization, allow successful reconstruction of large defects. The repair also highlights the importance of a highly collaborative multidisciplinary team, each contributing essential expertise.

## Disclosures

The authors report no conflict of interest concerning the materials or methods used in this study or the findings specified in this publication.

## Author Contributions

Primary surgeon: Feldstein. Assistant surgeon: Adapa, Simpson, Miller, Duron. Editing and drafting the video and abstract: Feldstein, Adapa, Tang, Schmoke, Duron. Critically revising the work: Feldstein, Adapa, Tang, Schmoke, Duron. Reviewed submitted version of the work: Feldstein, Adapa, Tang, Schmoke, Ring. Approved the final version of the work on behalf of all authors: Feldstein. Supervision: Feldstein, Adapa.

## Supplemental Information

### Patient Informed Consent

The necessary patient informed consent was obtained in this study.
